# Adjuvant chemotherapy with S-1 after curative chemoradiotherapy in patients with locoregionally advanced squamous cell carcinoma of the head and neck: Reanalysis of the ACTS-HNC study

**DOI:** 10.1371/journal.pone.0198391

**Published:** 2018-06-08

**Authors:** Akira Kubota, Eiji Nakatani, Kiyoaki Tsukahara, Yasuhisa Hasegawa, Hideki Takemura, Tomonori Terada, Takahide Taguchi, Kunihiko Nagahara, Hiroaki Nakatani, Kunitoshi Yoshino, Yuichiro Higaki, Shigemichi Iwae, Takeshi Beppu, Yutaka Hanamure, Kichinobu Tomita, Naoyuki Kohno, Kazuyoshi Kawabata, Satoshi Teramukai, Masato Fujii, ACTS-HNC Study Group

**Affiliations:** 1 Department Head and Neck Surgery, Kanagawa Cancer Center, Yokohama, Japan; 2 Translational Research Center for Medical Innovation, Foundation for Biomedical Research and Innovation, Kobe, Japan; 3 Department of Otolaryngology, Head and Neck Surgery, Tokyo Medical University Hachioji Medical Center, Hachioji, Japan; 4 Department of Head and Neck Surgery, Aichi Cancer Center Hospital, Nagoya, Japan; 5 Department of Otolaryngology, Yokohama Rosai Hospital, Yokohama, Japan; 6 Department of Otolaryngology, Hyogo College of Medicine, Nishinomiya, Japan; 7 Department of Otorhinolaryngology, and Head and Neck Surgery, Yokohama City University School of Medicine, Yokohama, Japan; 8 Department of Head and Neck Surgery, Kusatsu General Hospital, Kusatsu, Japan; 9 Division of Head and Neck Surgery, Tochigi Cancer Center, Utsunomiya, Japan; 10 Department of Otolaryngology, Head and Neck Surgery, Osaka Medical Center for Cancer and Cardiovascular Diseases, Osaka, Japan; 11 Department of Head and Neck Surgery, National Hospital Organization Kyushu Cancer Center, Fukuoka, Japan; 12 Department of Head and Neck Surgery, Hyogo Cancer Center, Akashi, Japan; 13 Department of Head and Neck Surgery, Saitama Cancer Center, Saitama, Japan; 14 Department of Otolaryngology, Kagoshima City Hospital, Kagoshima, Japan; 15 Department of Otolaryngology, Akasaka Surgery Clinic, Fukuoka, Japan; 16 Department of Otolaryngology, Head and Neck Surgery, Kyorin University School of Medicine, Mitaka, Japan; 17 Division of Head and Neck Surgery, Cancer Institute Hospital, Tokyo, Japan; 18 Department of Biostatistics, Kyoto Prefectural University of Medicine Graduate School of Medical Science, Kyoto, Japan; 19 Department of Otolaryngology, National Hospital Organization, Tokyo Medical Center, Tokyo, Japan; Tata Memorial Centre, INDIA

## Abstract

**Background:**

Chemoradiotherapy (CRT) has improved organ preservation or overall survival (OS) of locoregionally advanced head and neck squamous cell cancer (LAHNSCC), but in clinical trials of conventional CRT, increasing CRT intensity has not been shown to improve OS. In the Adjuvant ChemoTherapy with S-1 after curative treatment in patients with Head and Neck Cancer (ACTS-HNC) phase III study, OS of curative locoregional treatments improved more with adjuvant chemotherapy with S-1 (tegafur gimeracil oteracil potassium) than with tegafur/uracil (UFT). ACTS HNC study showed the significant efficacy of S-1 after curative radiotherapy in sub-analysis. We explored the efficacy of S-1 after curative CRT in a subset of patients from the ACTS-HNC study.

**Methods:**

Patients with stage III, IVA, or IVB LAHNSCC were enrolled in this study to evaluate the efficacy of S-1 compared with UFT as adjuvant chemotherapy after curative CRT in the ACTS-HNC study. Patients received S-1 at 80–120 mg/day in two divided doses for 2 weeks, followed by a 1-week rest, or UFT 300 or 400 mg/day in two or three divided doses daily, for 1 year. The endpoints were OS, disease-free survival, locoregional relapse-free survival, distant metastasis-free survival (DMFS), and post-locoregional relapse survival.

**Results:**

One hundred eighty patients (S-1, n = 87; UFT, n = 93) were included in this study. Clinical characteristics of the S-1 and UFT arms were similar. S-1 after CRT significantly improved OS (hazard ratio [HR], 0.46; 95% confidence interval [CI], 0.22–0.93) and DMFS (HR, 0.50; 95% CI, 0.26–0.97) compared with UFT.

**Conclusion:**

As adjuvant chemotherapy, S-1 demonstrated better efficacy for OS and DMFS than UFT in patients with LAHNSCC after curative CRT and may be considered a treatment option following curative CRT. For this study was not preplanned in the ACTS-HNC study, the results is hypothesis generating but not definitive.

## Introduction

Chemoradiotherapy (CRT) administered as curative or postoperative treatment for locoregionally advanced head and neck squamous-cell carcinoma (LAHNSCC) has been demonstrated to improve locoregional control, overall survival (OS), and organ preservation [1–10]. However, recent phase III studies that explored whether the combination of induction chemotherapy or molecularly targeted drug therapy and concurrent CRT enhances the treatment effectiveness of concurrent CRT failed to demonstrate improvement in OS [11–14]. Concurrent CRT with nonstandard fractionation schedules also failed to prolong OS [15, 16]. New therapeutic strategies to prolong the survival time of CRT have been investigated.

Although adjuvant chemotherapy after curative treatment for HNSCC has not been shown to improve OS [17–21], it has been reported to reduce the incidence of distant metastasis [17, 19, 21]. A previous report showed that tegafur/uracil (UFT) as adjuvant chemotherapy was preferable, in terms of rate of distant metastasis, to non-treatment after curative surgery in cases of HNSCC, but that there was no difference in disease-free survival (DFS) [21].

Like UFT, S-1 is an oral fluoropyrimidine preparation, but because it contains gimeracil, a dihydropyrimidine dehydrogenase inhibitor, S-1 has greater antitumor activity than UFT [22]. Indeed, S-1 as adjuvant chemotherapy after curative surgery of rectal cancers significantly increased the proportion of patients achieving relapse-free survival compared with UFT [23]. In the Adjuvant Chemotherapy with S-1 after definitive Treatment in Patients with Head and Neck Cancer (ACTS-HNC) study, OS was significantly longer with S-1 than with UFT [24]. If adjuvant therapy with S-1 after CRT can reduce the rate of distant metastasis and prolong OS in patients with LAHNSCC, S-1 may be of benefit.

Therefore, we examined the efficacy of S-1 as adjuvant chemotherapy after curative CRT. The present analysis compared OS, DFS, distant metastasis-free survival (DMFS), locoregional relapse-free survival (LRRFS), and post-LRR survival (post-LRRS) between S-1 and UFT among ACTS-HNC study (ClinicalTrial. gov: NCT00336947) patients who had undergone curative CRT.

## Methods

### Compliance with ethical standards

Because the current study was an explanatory analysis using the dataset of a previously reported clinical trial (ACTS-HNC study), no ethical approval was required.

### The ACTS-HNC study

Patients, study design, and methods of the ACTS-HNC study have been reported [24]. Briefly, patients with stage III, IVA, or IVB squamous-cell carcinoma of the maxillary sinus, oral cavity, oropharynx, hypopharynx, or larynx that disappeared, as confirmed by diagnostic imaging or biopsy, after a combination of curative treatments (radiotherapy or surgery) with or without chemotherapy were eligible for this trial. If residual tumor was suspected after curative therapies, additional treatment was followed. Additional treatment defined as either the addition of surgery to the curative radiotherapy ± chemotherapy, or the addition of radiotherapy ± chemotherapy to the curative surgery before random allocation. After completion of definitive treatment confirmed no residual tumor within 2 months, patients were randomly assigned (1:1) to receive either UFT or S-1. In the UFT group, patients received 300 mg/day [body surface area(BSA)<1.5 m^2^] or 400 mg/day (BSA≥1.5 m^2^) of UFT in two or three divided doses daily. In the S-1 group, patients received 80 mg/day (BSA<1.25 m^2^), 100 mg/day (BSA≥1.25 and <1.5 m^2^), or 120 mg/day (BSA≥1.5 m^2^) of S-1 in two divided doses for 2 weeks, followed by one week off treatment. Treatment duration for both arms was 1 year. Stratification factors for dynamic allocation included subset, stage (III, IVA, or IVB), type of curative therapy (surgery, radiotherapy, or both), and institution. Criteria for dose reduction were developed based on adverse events.

### Patient groups for analysis

**Fig 1:**
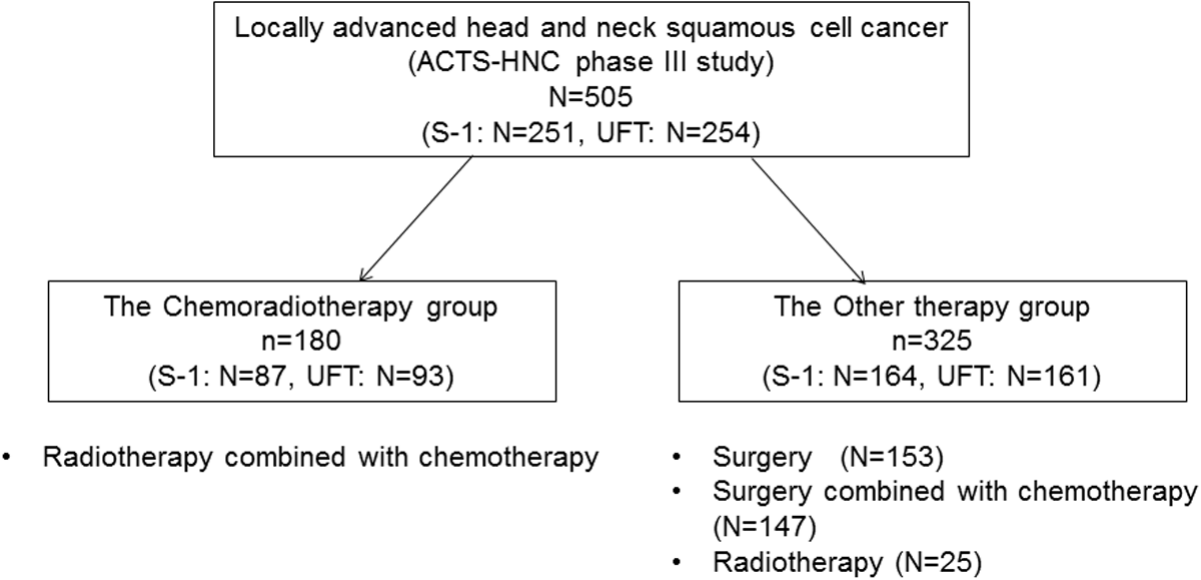
Subsets by curative treatment before random allocation. Abbreviations: ACTS-HNC, Adjuvant chemotherapy with S-1 after curative treatment in patients with Head and Neck Cancer. CRT, chemoradiotherapy.

Based on the type of curative treatments (surgery, radiotherapy), patients were divided into either the CRT group (concurrent or consecutive administration of chemotherapy with radiotherapy) or the Other Therapy group (surgery alone, surgery with chemotherapy, or radiotherapy alone) (Fig 1).

### Outcomes evaluated

OS, DFS, LRRFS, DMFS, and post-LRRS were evaluated in both groups. Of these, OS, DFS, post-LRRS were assessed in the phase III trial [24]. For the present study, additional outcomes were defined as follows: LRRFS was the time from the date of randomization to the first date of local recurrence, cervical lymph node recurrence, or disease-specific death. DMFS was the time to the first date of distant recurrence or distant metastasis in areas other than local or cervical lymph nodes, or death from any cause. The censoring time for DMFS was the date of the last observation that showed no recurrence. Post-LRRS was the time from the date of randomization to the date of disease-specific death after confirmed locoregional relapse (LRR). This censoring time was the date of the last observation indicating survival.

### Statistical analyses

This reanalysis was performed by using analysis dataset of ACTS-HNC study [24]. Clinical information was collected before randomization in the phase III trial. Clinical characteristics were compared using Fisher’s exact test for categorical variables such as sex (male or female), age (20–59, 60–69, or 70–75), performance status (0 or 1), subsite (maxillary sinus, oral cavity, oropharynx, hypopharynx, or larynx), stage (III, IVA, or IVB), and additional treatments. The Kaplan–Meier method was used to estimate OS, DFS, LRRFS, DMFS, and post-LRRS rates. Hazard ratios (HRs) and 95% confidence intervals (CIs) were estimated using Cox proportional-hazard models to evaluate effect size. The robustness of results for OS, DMFS, and post-LRRS was assessed by adjusting the effect of baseline characteristics in the log-rank test. Sex, age, performance status, subsite, stage, and additional treatment were added to the stratified log-rank test as stratification factors in the sensitivity analysis. Differences with a *P* value of <.05 were considered statistically significant. All statistical analyses were performed using SAS/STAT^®^ software, Version 9.3 (SAS Institute Inc., Cary, NC, USA) and R, Version 3.3.2 [25].

## Results

### Patient characteristics by curative therapy

Of the 505 ACTS-HNC study patients analyzed, 180 were in the CRT group (S-1 arm, n = 87; UFT, n = 93) and 325 were in the Other Therapy group (S-1 arm, n = 164; UFT, n = 161) (Table 1 and Fig 1).

**Table 1:** Comparison of clinical characteristics between the chemoradiotherapy group and the other therapy group.

Variable	Category	Chemoradiation (%)			Other therapy (%)			*P* value
		Total	S-1	UFT	Total	S-1	UFT	CRT vs. Others
**No. of patients**		180	87	93	325	164	161	
**Sex**	Male	155 (86)	73 (84)	82 (88)	269 (83)	136 (83)	133 (83)	0.3761
	Female	25 (14)	14 (16)	11 (12)	56 (17)	28 (17)	28 (27)	
**Age**	Median	62	61	63	61	62	61	0.1888
	Range	36–75	40–75	36–75	26–75	26–75	29–75	
**PS**	0	162 (90)	77 (89)	85 (91)	303 (93)	154 (94)	149 (93)	0.2289
	1	18 (10)	10 (11)	8 (9)	22 (7)	10 (6)	12 (7)	
**Stage**	III	55 (31)	27 (31)	28 (30)	78 (24)	40 (24)	38 (24)	0.0622
	IVA	115 (64)	55 (63)	60 (65)	238 (73)	121 (74)	117 (73)	
	IVB	10 (6)	5 (6)	5 (5)	9 (3)	3 (2)	6 (4)	
**Primary Site**	Maxillary Sinus	16 (9)	9 (10)	7 (8)	23 (7)	12 (7)	11 (7)	<0.001
	Oral Cavity	15 (8)	6 (7)	9 (10)	105 (32)	53 (32)	52 (32)	
	Oropharynx	55 (31)	22 (25)	33 (36)	53 (16)	31 (19)	22 (14)	
	Hypopharynx	71 (39)	37 (43)	34 (37)	71 (22)	34 (21)	37 (23)	
	Larynx	23 (13)	13 (15)	10 (11)	73 (22)	34 (21)	39 (24)	
**Additional Treatment**	Yes	53 (29)	30 (35)	23 (25)	199 (61)	102 (62)	97 (60)	<0.001
	None	127 (71)	57 (65)	70 (75)	126 (49)	62 (38)	64 (40)	

Data presented as Median [Range] or n (%).

Abbreviations: PS performance status.

### Comparisons between the CRT and Other Therapy groups and between S-1 and UFT in the CRT group

Comparisons of clinical characteristics between the CRT group and the other therapy group revealed significant differences in subsite (*P* <.001) and additional treatment (*P* <.001) (Table 1). However, baseline characteristics were generally similar between the S-1 and UFT arms within each group.

### Outcomes in the present study

**Fig 2:**
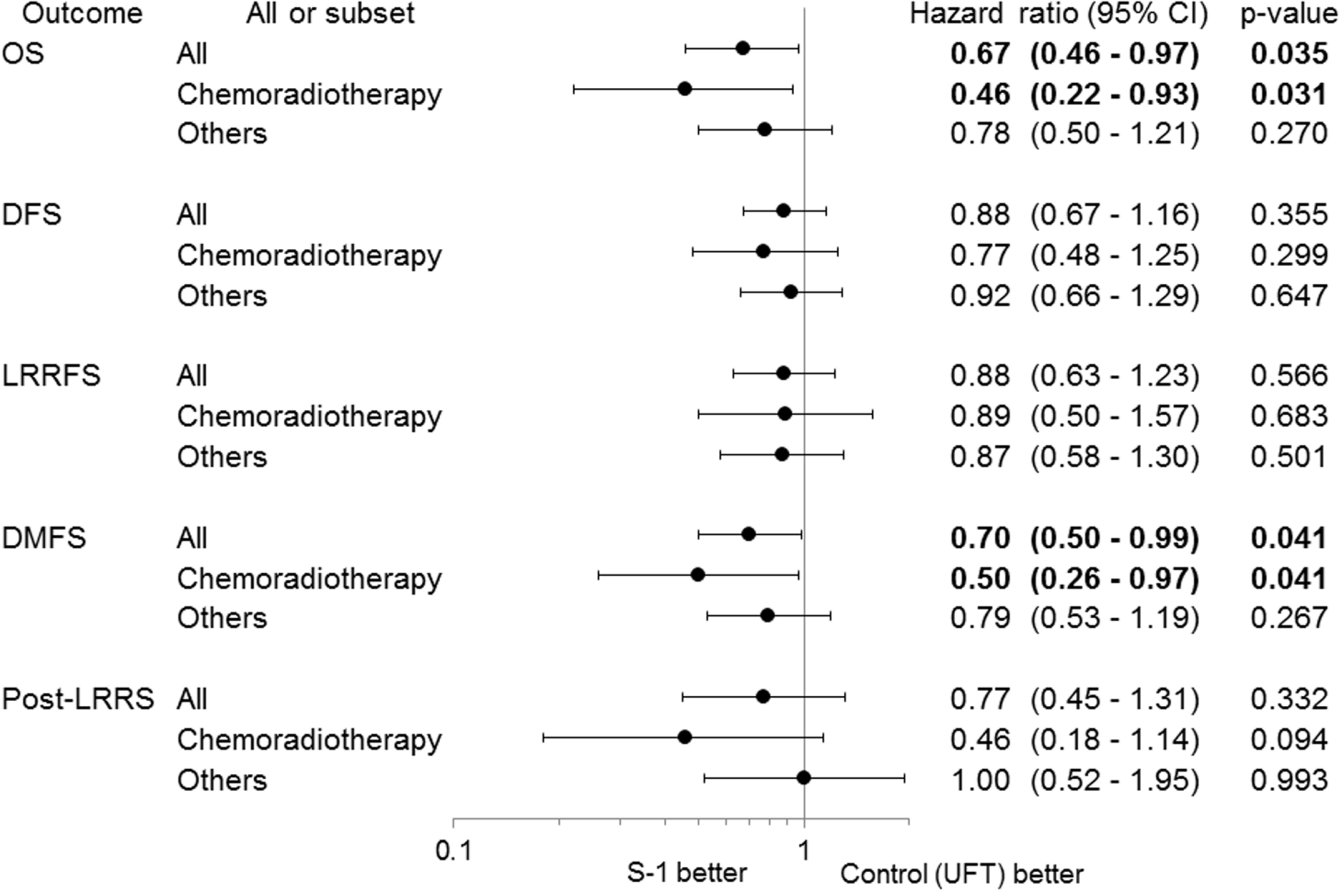
Forest plot for subset analysis. Abbreviations: CI, confidence interval. DFS, disease-free survival. DMFS, distant metastasis-free survival. LRRFS, locoregional relapse-free survival. OS, overall survival. post-LRRS, post-locoregional relapse-free survival. UFT, tegafur/uracil.

Median follow-up for all patients was 1356 days (range, 35–2116 days). The results of the analysis for OS, DFS, RFS, LRRFS, DMFS, and post-LRRS between the S-1 and UFT arms of the CRT group are presented in Fig 2.

### Overall survival

**Fig 3:**
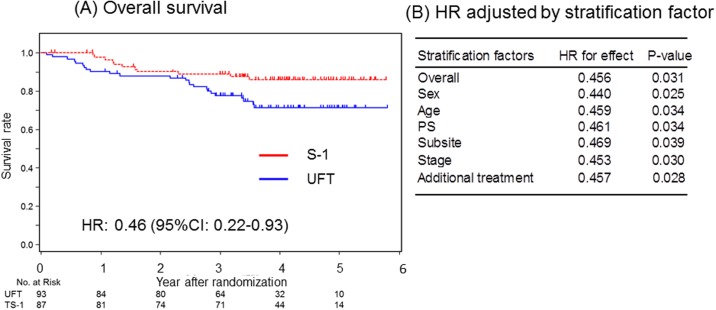
Overall survival in the CRT group. (A) OS derived from Kaplan–Meier curves. (B) HR and corresponding CI were calculated using Cox proportional hazard model. *P* values were calculated based on stratified log-rank test. Abbreviations: CI, confidence interval. HR, hazard ratio. OS, overall survival.

In the CRT group, S-1 significantly improved OS (HR, 0.46; 95% CI, 0.22–0.93; *P* = .031) (Fig 2A). Kaplan-Meier curves comparing the effect of S-1 and UFT on OS are shown in Fig 3A. In sensitivity analysis, the treatment effect of S-1 on OS remained significantly better after adjustment for all stratification factors (sex, age, performance status, subsite, stage, and additional treatment) (Fig 3B).

### Distant metastasis-free survival

**Fig 4:**
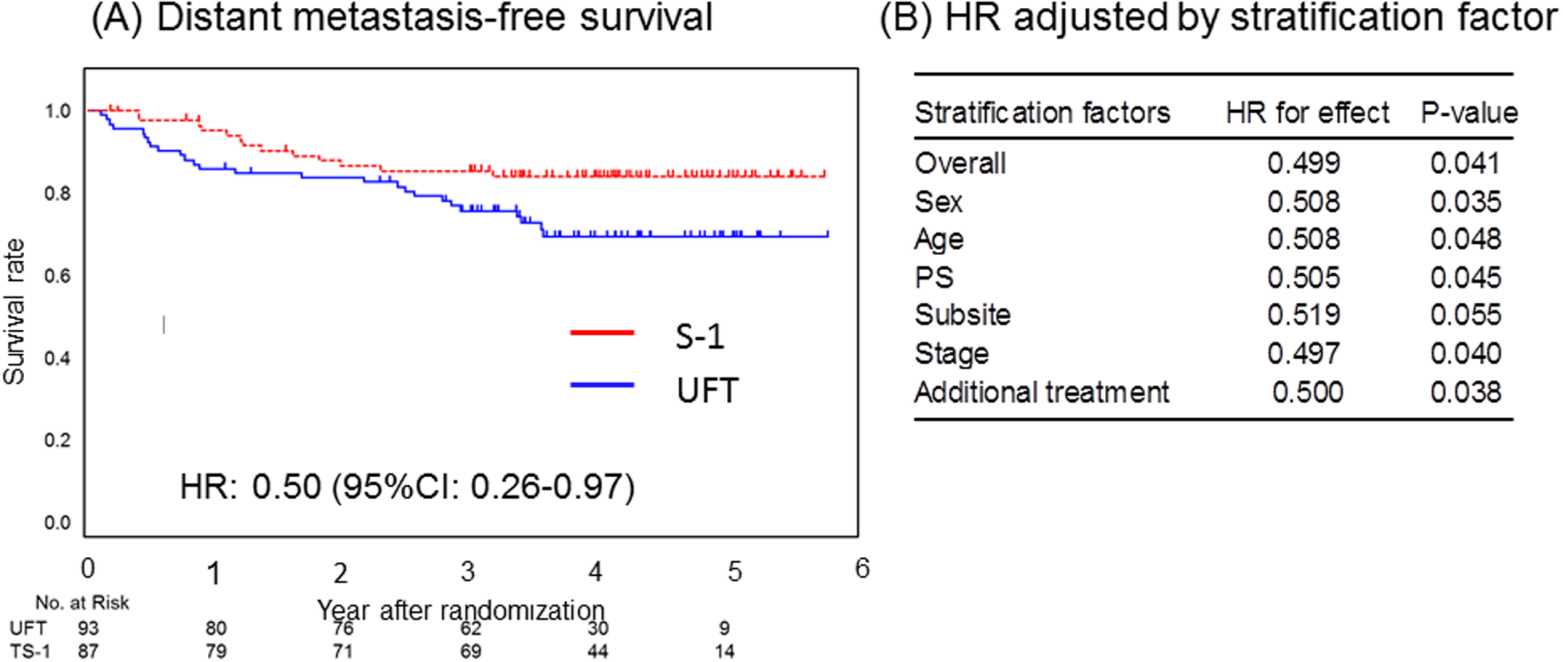
Distant metastasis-free survival in the CRT group. (A) DMFS derived from Kaplan–Meier curves. (B) HR and corresponding CI were calculated using Cox proportional hazard model. P values were calculated based on stratified log-rank test. Abbreviations: CI, confidence interval; HR, hazard ratio; DMFS, distant metastasis-free survival.

In the CRT group, S-1 significantly improved DMFS (HR, 0.50; 95% CI, 0.26–0.97; *P* = .041) (Fig 2A). Kaplan-Meier curves comparing the effect of S-1 and UFT on DMFS are shown in Fig 4A. In sensitivity analysis, when adjusting for stratification factors, the treatment effect of S-1 on DMFS remained significantly better excluding only after adjustment for subsite (Fig 4B).

### Post- locoregional relapse survival

**Fig 5:**
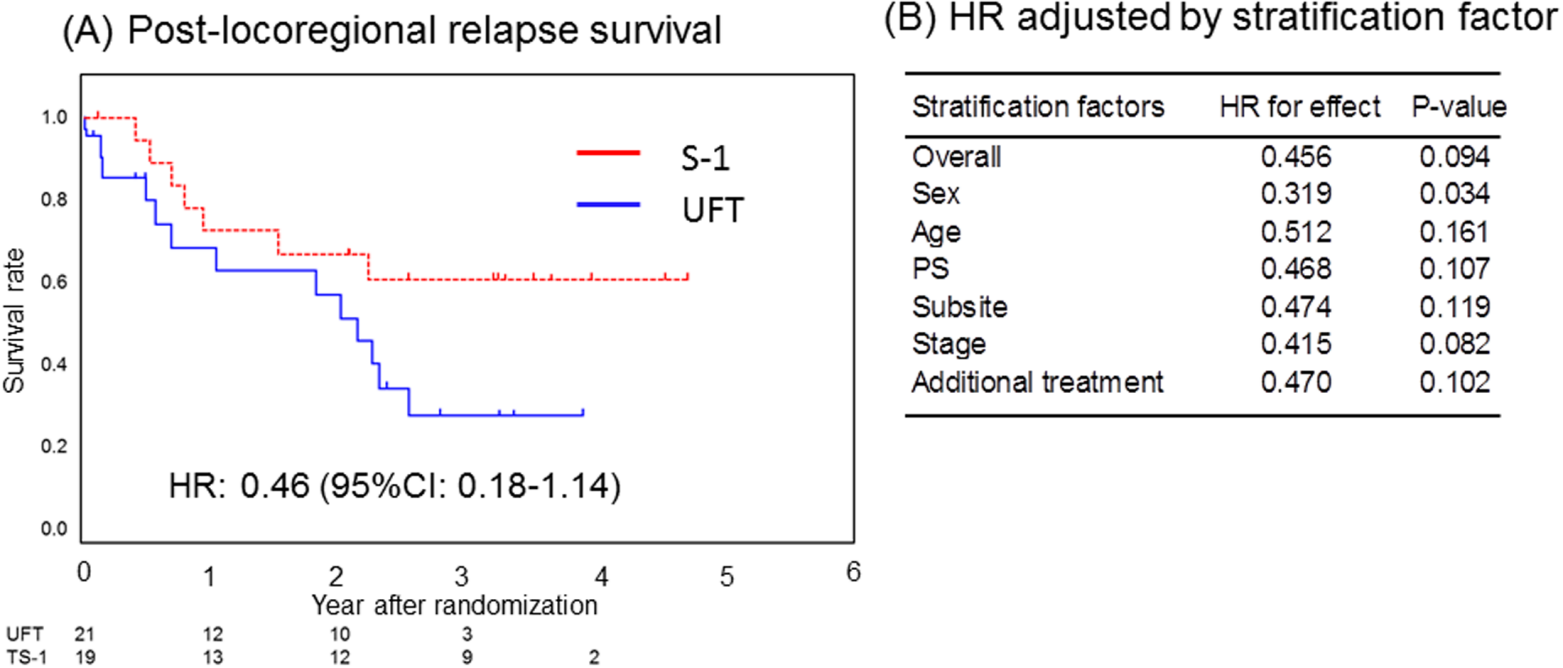
Post- locoregional relapse survival in the CRT group. (A) Post-LRRS derived from Kaplan–Meier curves. (B) HR and corresponding CI were calculated using Cox proportional hazard model. *P* values were calculated based on stratified log-rank test. Abbreviations: CI, confidence interval; HR, hazard ratio; post-LRRS, post-locoregional relapse-free survival.

In the CRT group, A trend was observed in improvement of post-LRRS by S-1, but the difference was not statistically significant (HR, 0.46, 95% CI, 0.18–1.14; *P* = .094) (Fig 2). Kaplan-Meier curves comparing the effect of S-1 and UFT on post-LRRS are shown in Fig 5A. In sensitivity analysis, when adjusting for stratification factors, the treatment effect of S-1 on post-LRRS remained significantly better only after adjustment for sex (Fig 5B).

### Disease-free survival and Locoregional relapse-free survival

DFS and LRRFS did not differ significantly between S-1 and UFT (Fig 2).

## Discussion

The present study has shown a survival effect of adjuvant chemotherapy with S-1 in a subset of patients who underwent CRT as initial curative treatment (CRT group) in a phase III trial. In the CRT group, adjuvant chemotherapy with S-1 was associated with significant improvement in OS and DMFS, and with a trend toward better post-LRRS, compared with UFT. These outcomes of OS and DMFS were also verified in all patients in the ACTS-HNC phase III study, and these significant improvements with S-1 in the CRT group remained after sensitivity analysis with adjustment of HR for stratification factors.

In the CRT group, 127 patients (71%) had no residual tumor without additional surgery. These patients might be consisted with responder of IC plus radiotherapy or concurrent CRT, since non-responders to IC or concurrent CRT would be recommended for surgical resection. In previous phase III studies, IC plus radiotherapy or concurrent CRT significantly reduced the number of occurrences of distant metastasis [1–3, 5, 10]. If reduced rate of distant metastasis was a suitable factor for evaluating the effectiveness of adjuvant chemotherapy, those patients who responded to IC or concurrent CRT would have further reduction of distant metastasis with adjuvant chemotherapy following CRT. Indeed, the rate of distant recurrence in the CRT group was significantly lower than that of the Other Therapy group (*P* = .044) (data not shown) and, in addition to decreasing the rate of distant recurrence, S -1 displayed a survival effect more clearly than did UFT.

Although locoregional control was similar between the S-1 and UFT arms, S-1 showed a greater trend toward better post-LRRS in the CRT group, which may further explain the efficacy of S-1. There have been no phase III studies confirming an OS benefit from adjuvant chemotherapy [17–21]. Three studies [17, 19, 21] indicated that adjuvant chemotherapy reduced distant metastasis in patients undergoing curative surgery, and in two of the studies [17, 19], surgery plus radiotherapy was considered definitive treatment. Definitive treatment after LRR is surgery or radiotherapy; after surgery, postoperative radiotherapy with or without chemotherapy is recommended, so that salvage treatment of LRR after curative surgery is limited and will not improve OS. In contrast, because many patients in the CRT group had no residual tumor without additional surgery, salvage surgery after LRR might be an option. In addition, the curability of salvage surgery might have been increased by S-1 more than by UFT because it limited the extent of recurrence. Future clinical studies of adjuvant chemotherapy must incorporate not only salvage therapy following LRR, but also patient follow-up, in their designs.

It has been reported that the prognosis of oropharyngeal cancer is improving because oropharyngeal cancer related to human papillomavirus (HPV) responds well to chemotherapy and radiotherapy, but that HPV-related oropharyngeal cancer has been increasing over the years [26, 27]. It has also been reported that the prognosis in patients with HPV-related oropharyngeal cancer is well preserved in nonsmokers and that smoking is a poor prognostic factor. In the present study, data on HPV expression and smoking status were not available for patients with oropharyngeal cancer. However, it is unlikely that the lack of information on HPV status contributed to better results with S-1 because fewer patients with oropharyngeal cancer were treated with S-1 than with UFT.

The present study may be limited by the possibility of statistical error from ad hoc analysis or subset analysis from trial data because what we studied here was not preplanned in the ACTS-HNC study. Caution should therefore be exercised, and readers should be aware that the study is only an exploratory subset analysis. We were unable to determine the proportions of patients who received induction versus concurrent CRT. However, no previous study of adjuvant chemotherapy shown improvement in the OS of curative locoregional treatment or curative CRT; therefore, the outcomes of this study, that S-1 as adjuvant chemotherapy after curative CRT may improve OS and DMFS and possibly ameliorate poor prognosis of LAHNSCC, are still important. Clinical trials that clarify the efficacy of S-1 as adjuvant chemotherapy after curative CRT should be encouraged.

## Conclusion

The results of the present study suggest that adjuvant chemotherapy with S-1 may improve OS and DMFS compared with UFT in patients who have received CRT as curative treatment that resulted in no residual tumor. Adjuvant chemotherapy with S-1 may therefore be considered a treatment option following CRT.

## Abbreviations

ACTS-HNC: Adjuvant Chemotherapy with S-1 after curative treatment in patients with Head and Neck Cancer
BSA: body surface area
CI: confidence interval
CRT: chemoradiotherapy
DFS: disease-free survival
DMFS: distant metastasis-free survival
HPV: human papillomavirus
HR: hazard ratio
LAHNSCC: locoregionally advanced head and neck squamous cell cancer
LRR: locoregional relapse
LRRFS: locoregional relapse-free survival
OS: overall survival
post-LRRS: post-LRR survival
UFT: tegafur/uracil
